# Rib Plating of Recurrent, Nonunion Rib Stress Fractures in an Elite Athlete

**DOI:** 10.1016/j.atssr.2026.03.011

**Published:** 2026-04-01

**Authors:** Elaine Liang, Michael Ou, Jared Peter C. Sarinas, Amanda Soe, Adrian Valderrama, Barkha Trivedi, Ivana Vasic, Jeffrey B. Velotta

**Affiliations:** 1 Kaiser Permanente Bernard J. Tyson School of Medicine, Pasadena, California; 2 Department of Surgery, University of California, San Francisco, San Francisco, California; 3 University of California, San Francisco, School of Medicine, San Francisco, California; 4 Department of Surgery, University of California, San Francisco, East Bay, Oakland, California; 5 University of California, Los Angeles, Los Angeles, California; 6 Department of Thoracic Surgery, Kaiser Permanente Oakland Medical Center, Oakland, California

## Abstract

Rib plating is well established for severe traumatic rib fractures, but its application in rib stress fractures (RSFs) remains poorly defined. We report the case of a Division I female collegiate rower presenting with recurrent anterior thoracic RSFs and pain refractory to conservative management. She underwent rib plating with chest wall reconstruction by a local muscle flap, with complete pain resolution reported 4 weeks postoperatively. This case suggests that for select patients, particularly those exposed to high thoracic stress loading, rib plating may represent an effective treatment strategy for refractory RSF-related pain.

Rib stress fractures (RSFs) result from repetitive mechanical loading and are commonly observed in athletes performing repetitive upper body motion, such as rowers.^1^^,^^2^ RSF occurs in 8.1% to 16.4% of elite rowers^1^ and represents the leading cause of time lost from rowing.^3^ Persistent stress or inadequate stabilization may disrupt healing and lead to nonunion, in which the rib fails to rejoin, leading to chronic chest wall pain, exertional dyspnea, and functional limitation.^1^^,^^4^ Despite its prevalence, RSF is poorly understood and frequently misdiagnosed as intercostal muscle strain, delaying diagnosis and return to sport.^3^^,^^5^ Current management guidelines emphasize conservative treatment, including cessation of rowing, analgesia, ultrasound or laser treatment, and staged return to sports with biomechanical optimization and technical assessment of rowing technique.^5^Although surgical fixation with rib plating and bone grafting has demonstrated efficacy for symptomatic rib nonunion after traumatic injury,^2^^,^^4^^,^^6^ its protective role in preventing further stress fractures in elite athletes remains poorly defined. We report a case of a Division I female collegiate rower with recurrent left fifth and sixth RSFs refractory to extensive conservative management, highlighting the role of surgical stabilization in restoring function and preventing recurrence.

A 20-year-old elite female rower presented with a history of recurrent left fifth and sixth RSFs causing debilitating pain since high school. She sustained 3 confirmed fractures with multiple suspected fractures over the years, culminating in a symptomatic nonunion of the left fifth rib after injury at national competitions despite conservative treatment. She was otherwise healthy (height, 5′11″; weight, 161 pounds; body mass index, 22.5 kg/m^2^), with no evidence of osteoporosis, hormonal abnormalities, or relevant genetic history. Conservative management, including physical therapy, strength training, and rest, failed to prevent recurrence. The rib fractures have significantly affected her athletic performance. Chest magnetic resonance imaging and computed tomography with contrast enhancement demonstrate chronic nonunion of the left sixth rib, with subtle callus formation consistent with a healing nondisplaced fracture, and an acute fracture of the left fifth rib (Figure 1). Pulmonary function test results and bone density assessment findings were normal.

**Figure 1:**
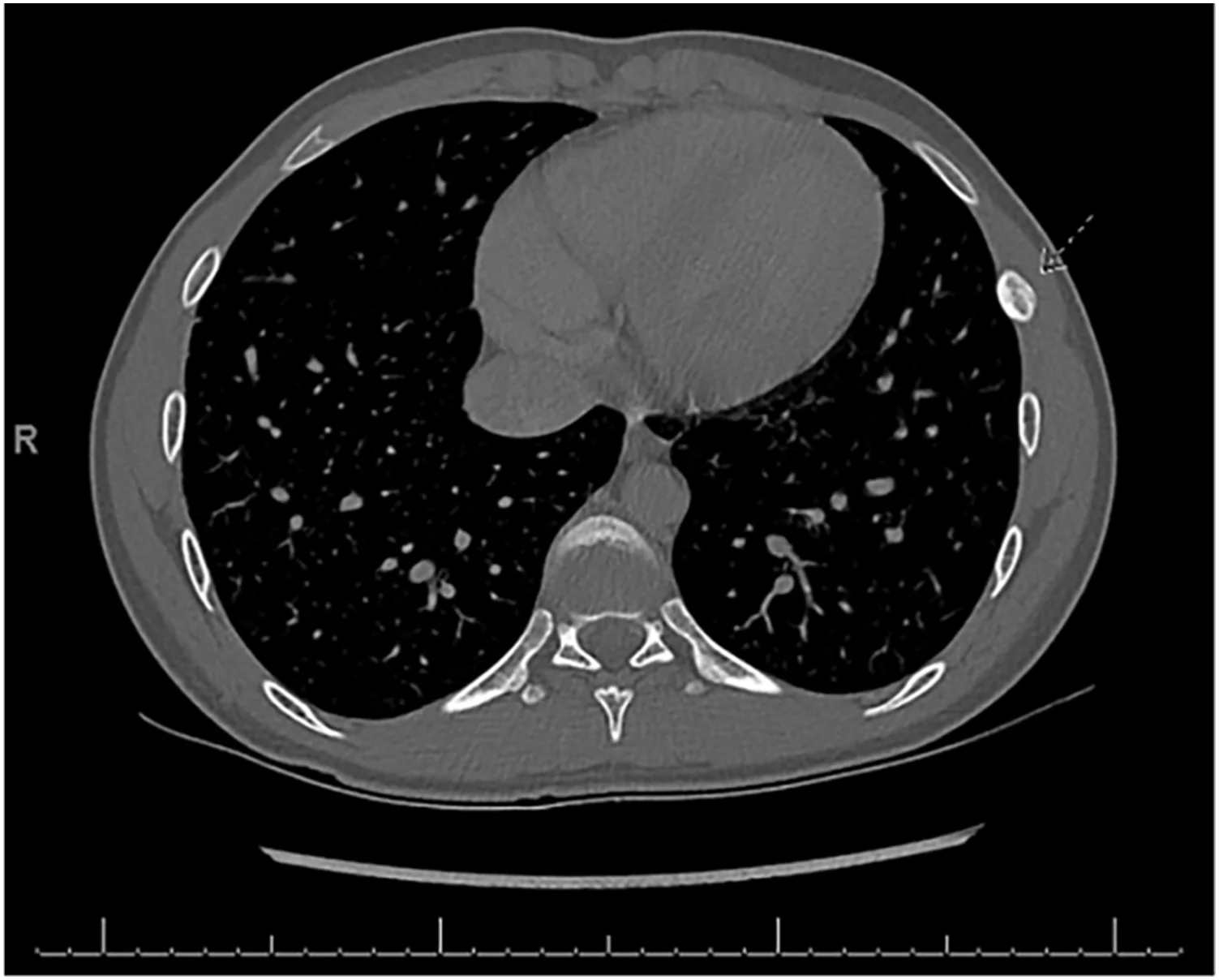
Computed tomography of the chest of T5 and T6 fractures. The arrow shows the fracture. (R, right [part of the CT scan].)

After multidisciplinary consultation, she underwent open reduction and internal fixation of the left fifth and sixth ribs with rib plating and chest wall reconstruction by a local muscle flap. Bone grafting was not instilled because the nonunion RSF was relatively small and for avoidance of additional morbidity associated with a donor graft site. Under general anesthesia, we performed muscle-sparing flap reconstruction by creating a lateral inframammary fold incision and elevated serratus anterior muscle flaps to expose the ribs. The anterior rib surface was marked with methylene blue (Figure 2A). Subperiosteal dissection was performed to fully expose the affected rib segments. The ribs were noted to be healing, with a callus formed at the sixth rib and a small, healed stress fracture at the fifth rib (Figure 2B).

**Figure 2:**
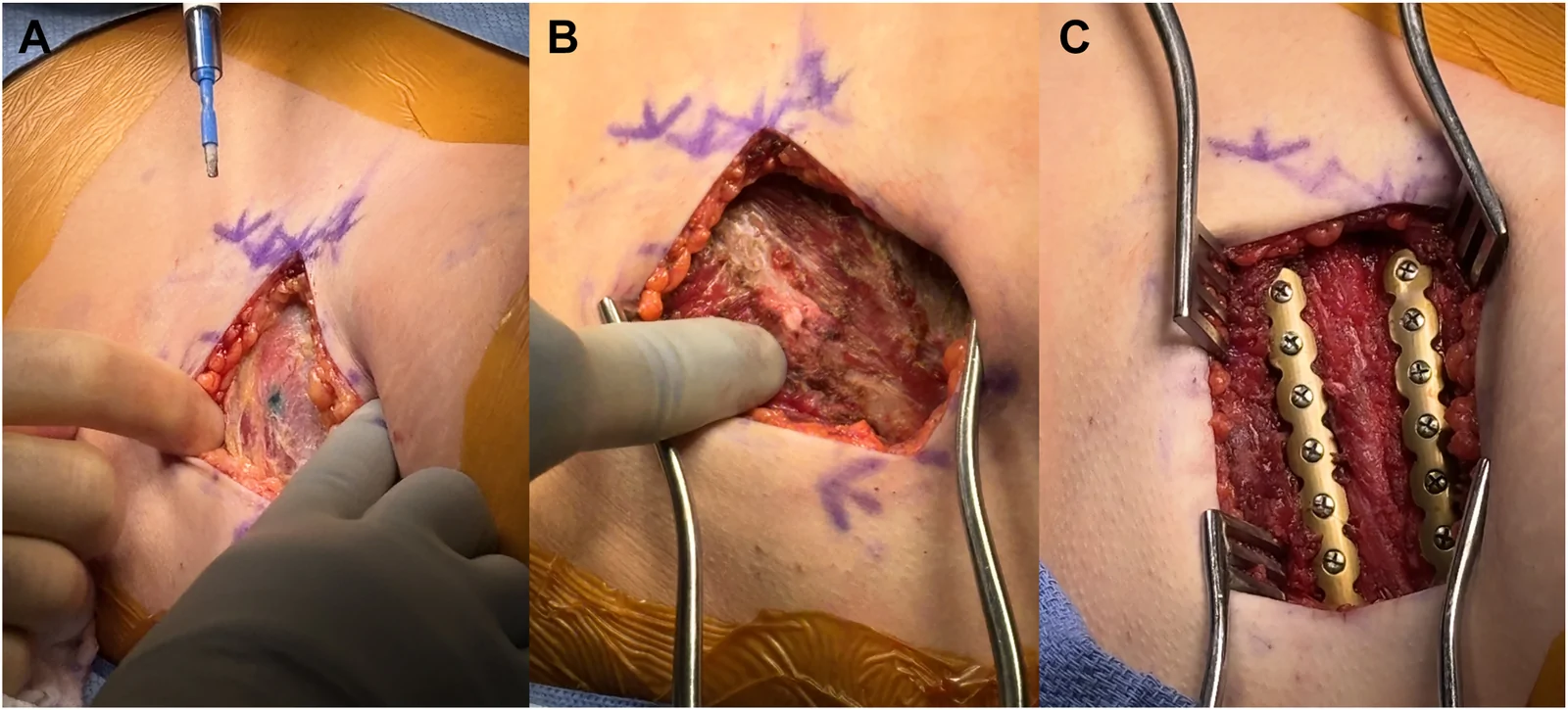
Intraoperative view. (A) Rib marked with methylene blue. (B) Healed stress fracture. (C) Final placement of two 8-hole Synthes plates over the left fifth and sixth ribs.

Two 8-hole Synthes plates were applied across the fifth and sixth ribs, securing them with 8-mm and 9-mm screws on both medial and lateral sides of the plates. Screw lengths were premeasured with calipers to ensure appropriate fixation, and the plates were positioned centered over the fracture sites, sitting flush with the anterior-superior rib surfaces without any outpouching (Figure 2C).

Cryoablation was performed at 3 intercostal levels (above fifth rib, between fifth and sixth ribs, and below sixth rib) for 1 minute and 30 seconds at each level for postoperative pain control. Liposomal bupivacaine was infused into the muscle and soft tissue layer to further optimize analgesia. After rib fixation, the serratus anterior was closed in 2 layers, repairing the underside and suturing it back down to the intercostal muscles. There were no postoperative complications; the patient was discharged on postoperative day 0 and is doing well. At 1-month and 6-month follow-up, chest radiographs demonstrated intact plate alignment, and the patient no longer requires analgesic medications. She is rowing at full capacity, with no symptoms currently (Figure 3).

**Figure 3:**
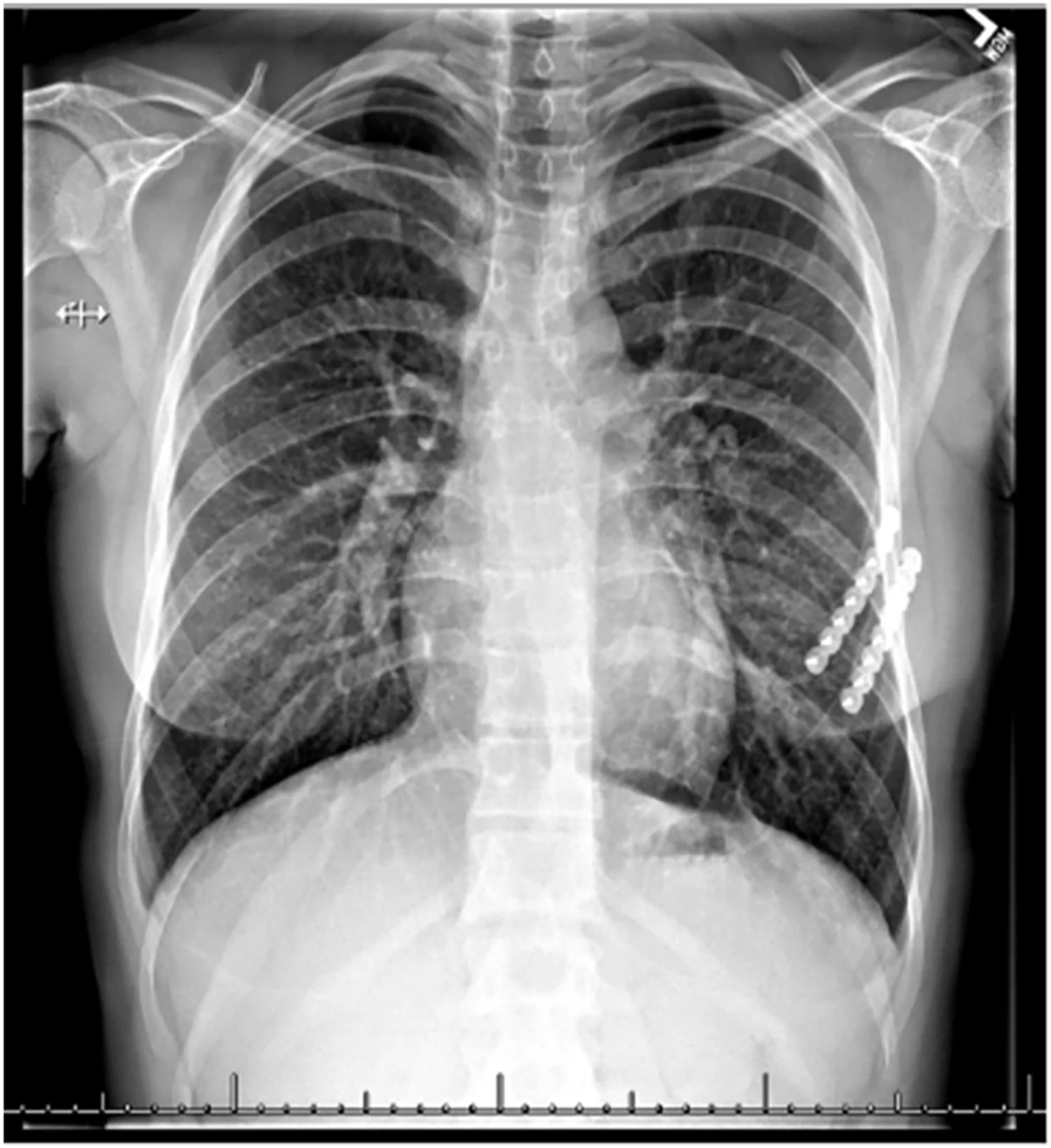
Postoperative chest radiograph of rib plates.

## Comment

Although open reduction and internal fixation with rib plating are well established for severe traumatic rib fractures and chronic nonunion,^6^ evidence supporting prophylactic use in elite athletes with high rib cage demands remains limited. In the general population, DeGenova and colleagues^7^ demonstrated that surgical fixation of traumatic rib fractures reduced pain and narcotic use, improved fracture union, and was associated with low complication rates.

In contrast, sports-related RSFs have traditionally been managed conservatively with rest, activity restriction, and analgesia, with gradual return to sport, symptoms permitting.^2^ Thus, current guidelines largely do not include surgery in RSF management.^3^^,^^5^ However, for elite athletes with persistent symptoms or nonunion despite exhaustive conservative treatment, surgical intervention may represent a viable option to restore function and to facilitate return to competition.

Reports of operative management for RSF in athletes are scarce and have primarily involved rib resection in the setting of thoracic outlet syndrome.^2^^,^^8^ Fearing and colleagues^2^ described successful open reduction and internal fixation with muscle-sparing technique and autologous bone grafting in an elite tennis player, resulting in full recovery and return to competition.

In conclusion, our case demonstrates novel use of rib plating as a prophylactic and reconstructive intervention in an elite rower with recurrent RSF and nonunion. This experience suggests that in carefully selected high-performance athletes who fail to respond to conservative therapy, open reduction and internal fixation may offer durable stabilization, prevent recurrent injury, and allow safe return to high-level sport.
